# Impact of SGLT2 Inhibitors on Magnesium in Kidney Transplant Patients with and Without Diabetes

**DOI:** 10.3390/ijms26072904

**Published:** 2025-03-22

**Authors:** Carmine Secondulfo, Nicoletta Vecchione, Dora Russo, Sarah Hamzeh, Candida Iacuzzo, Luca Apicella, Renata Angela Di Pietro, Antonio Pisani, Maria Amicone, Massimo Cirillo, Giancarlo Bilancio

**Affiliations:** 1 Department of Medicine, Surgery and Dentistry “Scuola Medica Salernitana”, University of Salerno, 84081 Baronissi, Italy; csecondulfo@unisa.it (C.S.); mcirillo@unisa.it (M.C.); 2 Department of Public Health, University of Naples “Federico II”, 80131 Naples, Italy; nicolettavecchione@gmail.com (N.V.); sarahhamzeh1@gmail.com (S.H.); antonio.pisani13@gmail.com (A.P.); ma.amicone.90@gmail.com (M.A.); 3 Unit of Nephrology, Dialysis and Transplant, Salerno University Hospital “San Giovanni di Dio e Ruggi d’Aragona”, 84131 Salerno, Italy; candida.iacuzzo@sangiovannieruggi.it (C.I.); luca.apicella@sangiovannieruggi.it (L.A.); angela.dipietro@sangiovannieruggi.it (R.A.D.P.)

**Keywords:** SGLT2i, dapagliflozin, serum magnesium, kidney transplant

## Abstract

Magnesium (Mg^2+^) is essential for cardiovascular and metabolic health, yet hypomagnesemia is common in kidney transplant recipients (KTRs) due to immunosuppressive therapy and renal dysfunction. Oral Mg^2+^ supplementation is often ineffective due to poor absorption and side effects. Sodium-glucose cotransporter 2 inhibitors (SGLT2i) have been shown to increase serum Mg^2+^ in chronic kidney disease, but their effects in KTRs, particularly patients without diabetes, remain unclear. This observational study assessed 63 KTRs treated with dapagliflozin, analyzing the serum Mg^2+^ levels at baseline and after 3 and 6 months. The hypomagnesemia prevalence, associations with oral supplementation, diabetes status, and diuretic use were evaluated. The results showed a significant Mg^2+^ increase with SGLT2i therapy, reducing hypomagnesemia regardless of the diabetes status. Oral supplementation did not correlate with improved Mg^2+^ levels, reinforcing its limited efficacy. Additional benefits included reductions in the body weight, blood pressure, and serum urate without compromising graft function. SGLT2i may offer a novel approach to managing hypomagnesemia in KTRs, potentially reducing the reliance on ineffective supplements while providing renal and cardiovascular benefits. Further research is needed to confirm these findings and elucidate the underlying mechanisms.

## 1. Introduction

Magnesium (Mg^2+^) is an electrolyte that plays a fundamental role in human health. Mg^2+^ is crucial for maintaining cardiac excitability, regulating blood pressure, preserving bone integrity, supporting glucose and insulin metabolism, and modulating the immune system [1,2]. Acute hypomagnesemia can lead to the rapid onset of potentially lethal events, such as torsades de pointes, neuromuscular paralysis, seizures, and coma [3]. In the long term, Mg^2+^ deficiency has been linked to an increased risk of cerebrovascular events, hypertension, cardiovascular disease, type 2 diabetes, and osteoporosis [4]. Furthermore, chronic hypomagnesemia is associated with an increased risk of atherosclerosis, depression, schizophrenia, Parkinson’s disease, and dementia [3]. Low serum Mg^2+^ is associated with adverse clinical outcomes and increased mortality both in the general population [5,6,7] and in chronic kidney disease (CKD) patients [8,9]. While direct causation is still debated, chronic low serum magnesium is also a strong predictor of CKD progression in the long term [8].

Hypomagnesemia is a frequent electrolyte imbalance in kidney transplant recipients (KTRs), often attributed to calcineurin inhibitor (CNi) therapy, and tacrolimus in particular [10,11,12,13]; chronic diarrhea, which is a common adverse effect of mycophenolate, can lead to low magnesium absorption; tubular atrophy and concomitant use of diuretics, which are common in KTRs, can also contribute to renal wasting of this fundamental ion [14]. Given these considerations, Mg^2+^ represents an important modifiable risk factor in this especially vulnerable category of patients.

Many strategies are currently available to treat hypomagnesemia. The avoidance of drugs interfering with Mg^2+^ metabolism is the first step, but in KTRs, this is often unfeasible: CNis and mycophenolate are cornerstones of immunosuppressive therapy, and therapy conversion carries the possible risk of increased rejection [15].

Oral supplementation can also represent a feasible option. However, conventional oral magnesium supplementation, regardless of the preparation used, exhibits poor intestinal absorption, leading to limited bioavailability and reduced effectiveness. Additionally, it is often associated with adverse gastrointestinal side effects [16].

Sodium-glucose cotransporter type 2 inhibitors (SGLT2i) have shown an additional beneficial effect on serum Mg^2+^ levels in CKD patients [17,18]. SGLT2i are commonly prescribed in CKD, but data are lacking in the context of kidney transplants, and especially in KTRs without diabetes. This observational study reports the effect on magnesium balance in KTRs both with and without diabetes.

## 2. Results

A total of 63 kidney transplant patients were enrolled, of whom 82.5% were males, with a median age of 55 (IQR 41.8–55) years and a mean estimated glomerular filtration rate (eGFR) of 56 ± 19 mL/min/1.73 m^2^. The complete baseline characteristics are shown in Table 1.

Twenty-four patients (38.1%) in our cohort showed low serum magnesium at baseline (T0). A proportion of 50% of these subjects had ongoing oral supplementation, while the remaining 50% did not take any supplementation, despite previous prescription, due to the adverse gastrointestinal effects of oral preparations or economic burden.

Low magnesium was not associated with the presence of diabetes and showed no significant difference in patients in therapy with cyclosporine or tacrolimus. A negative significative association was found between hypomagnesemia and concurrent diuretic therapy (*p* = 0.029).

**Figure 1:**
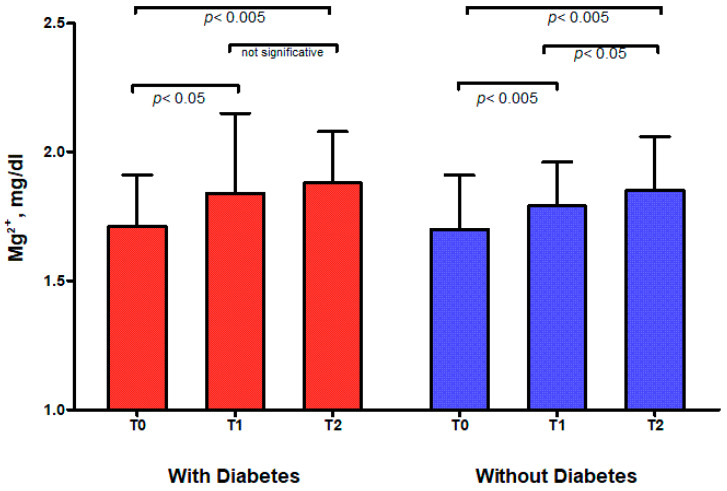
Three- and six-month trend in serum magnesium for KTRs with and without diabetes.

Serum magnesium increased significantly after 3 (*p* < 0.001) and 6 (*p* = 0.039) months of therapy with dapagliflozin in the whole cohort, and for subjects both with and without diabetes (Figure 1 and Table 2), with a significant concurrent reduction in the prevalence of hypomagnesemia (Table 3). As shown in Figure 1, the KTRs without diabetes showed no further relevant improvement in magnesemia at T2 in comparison to T1.

**Figure 2:**
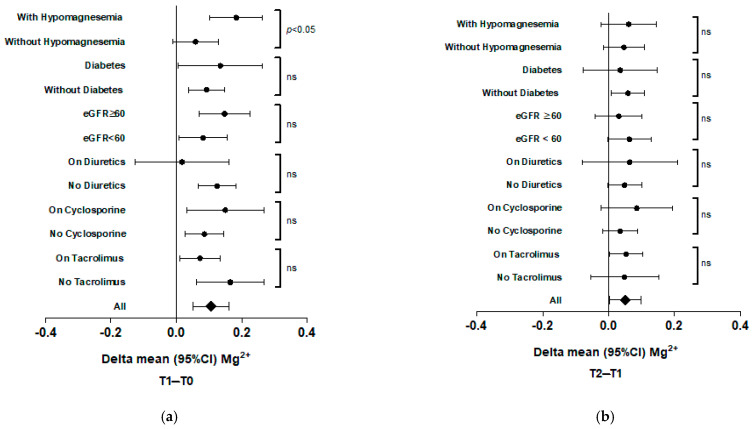
Difference in delta mean serum Mg^2+^ in subgroups of patients. (**a**) T1-T0; (**b**) T2-T1. (ns = not significative).

No significative differences were found in the delta mean of magnesemia between T1-T0 and T2-T1 in the baseline characteristics of the subgroups of patients, with the only exception being the subjects with and without hypomagnesemia (*p* = 0.02) in the T1-T0 comparison (Figure 2). In the KTRs who had overt hypomagnesemia at T0, the positive delta mean Mg^2+^ levels were higher.

A statistically significant reduction in body weight, systolic and diastolic blood pressure, serum urate, and eGFR were observed between T1 and T0 (Table 4); no further differences, aside from the aforementioned change in serum magnesium, were found for any of the other variables between T2 and T1.

## 3. Discussion

Hypomagnesemia is prevalent in the examined KTR population.

Oral supplementation with Mg^2+^ was commonly prescribed in the KTRs with low serum magnesium, but ongoing therapy was not associated with a lower prevalence of hypomagnesemia. This is in accordance with the current literature, as magnesium therapy is known to be vastly ineffective [16].

In the examined KTR cohort, an increase in serum magnesium was observed after SGLT2i therapy initiation for both the patients with and without diabetes. This finding is in accordance with a previous study by Sánchez Fructoso et al. on 339 KTRs with diabetes [19] and by Song et al. in a cohort of 50 KTRs with diabetes [20]. While the increase in the serum Mg^2+^ in subjects with diabetes undergoing SGLT2i therapy is well documented [21], there are no data in the current literature regarding SGLT2i’ effects on the serum Mg^2+^ in KTRs without diabetes.

SGLT2i are now considered part of the “standard of care” in CKD patients; in KTRs, however, the data are currently limited to subjects with diabetes only, while the potential benefit of this class of drugs in KTRs without diabetes is underexplored.

Hypomagnesemia was found to be more frequent in patients without concurrent diuretic therapy; this is in contrast with the current knowledge, as furosemide and thiazides are known to cause urinary magnesium loss [22,23,24]. It is possible that the CNi magnesium wasting effect could outweigh diuretic-induced hypomagnesemia in KTRs.

There are several benefits of restoring serum Mg^2+^ in kidney patients: hypomagnesemia is associated with hypertension, cardiovascular disease and cerebrovascular events, and osteoporosis [4]; some authors even suggest a pre-emptive initiation of SGLT2i therapy in order to prevent the development of PTDM in KTRs [20]. Additionally, low serum magnesium in KTRs is associated with accelerated decline in kidney function and with delayed graft function; hence, correcting the serum Mg^2+^ levels could be crucial for KTRs [13].

The potential for SGLT2i to enhance magnesemia presents a clinically relevant opportunity to improve post-transplant metabolic stability and overall patient outcomes. By mitigating magnesium depletion, SGLT2i could reduce the reliance on oral supplementation, leading to better adherence and fewer side effects while simultaneously offering their well-documented renal and cardiovascular benefits. Given the limited efficacy of currently available oral magnesium supplements and their frequent gastrointestinal side effects [16], many transplant patients struggle to achieve adequate magnesium levels. In Italy, these supplements are also not reimbursed, placing an additional financial burden on patients.

The major strength of this study is the inclusion of KTRs without diabetes, an absolute novelty in the current literature. However, this study has some limitations, including a relatively short follow-up period, its single-center design, the lack of a control group, and its retrospective, observational nature.

## 4. Materials and Methods

This analysis is part of the “Salerno CKD Cohort Study”, an ongoing, open-ended observational study encompassing the entire spectrum of chronic kidney disease, including kidney transplant recipients [25]. The study was reviewed and approved by the local institutional ethics committee (2012—n. 589) and conducted in accordance with the World Medical Association’s Code of Ethics (Declaration of Helsinki). It included written informed consent and was registered in the public databases of both the Italian Drug Agency (Agenzia Italiana del Farmaco, AIFA, ID n. 654) and the Authority for Privacy of the Italian Parliament (Garante della Privacy, n. 102400183803).

The inclusion criteria for this analysis were as follows: age ≥ 18 years, post-transplant duration of at least one year, proteinuria while on a stable treatment regimen for at least 12 weeks with renin–angiotensin system inhibitors at the maximum tolerated dose, and an estimated glomerular filtration rate (eGFR) ≥ 25 mL/min/1.73 m^2^, as per the requirements of the Italian Drug Agency [26]. eGFR was calculated using CKD-Epi equation [27].

Eligible patients were prescribed 10 mg/day of dapagliflozin in addition to their existing treatment regimen. Clinical and laboratory assessments were conducted at baseline (T0) (before treatment initiation) and after three (T1) and six months (T2) of dapagliflozin therapy.

The collected data included serum magnesium, albumin, and creatinine levels, along with self-reported gender, patient age, transplant age at enrollment, body weight, sitting blood pressure, ongoing antihypertensive and antidiabetic treatments, serum creatinine, and serum glucose levels. Hypomagnesemia was defined as serum magnesium < 1.7 mg/dL [28]. Routinely, patients with hypomagnesemia are prescribed oral Mg^2+^ supplementation. To avoid any potential bias, ongoing Mg^2+^ oral supplementation was noted, and no changes in Mg^2+^ therapy were made during the observation period.

Descriptive statistics were reported as the prevalence of categorical variables, mean ± SD of non-skewed numerical variables, and median with inter-quartile range (IQR) of numerical skewed variables (skewness > 1). The statistical analyses included the *t*-test for numerical non-skewed variables, the Wilcoxon and Mann–Whitney test for numerical skewed variables, Pearson chi-squared test for categorial variables, and McNemar test for categorial paired data. Results were considered statistically significant for *p* values ≤ 0.05. Statistical analyses were performed using SPSS 19 (IBM, Armonk, NY, USA).

## 5. Conclusions

SGLT2i are effective at increasing the serum magnesium levels during a 6-month treatment period in kidney transplant recipients both with and without diabetes, offering an additional but critical benefit, while simultaneously offering renal and cardiovascular benefits.

## Figures and Tables

**Table 1:** Baseline (T0) characteristics.

Number of patients	63
Sex, male, *n* (%)	52 (82.5%)
Age, years (median, IQR)	55 (41.8–55)
Diabetes, *n* (%)	20 (31.7%)
IFG	8 (12.7%)
Weight, kg	81.8 ± 15.7
SBP, mmHg	139 ± 15
DBP, mmHg	83 ± 10
Hypertension	63 (100%)
Diuretic therapy	11 (17.5%)
Mg^2+^ supplements	21 (33.3%)
Dyslipidemia	56 (89.9%)
Tacrolimus, *n* (%)	40 (63.5%)
Ciclosporine, *n* (%)	20 (31.7%)
Everolimus, *n* (%)	15 (23.8%)
Mycophenolate, *n* (%)	44 (69.8%)
Steroids, *n* (%)	60 (95.2%)
Creatinine, mg/dL	1.45 ± 0.45
eGFR mL/min/1.73 m^2^	56 ± 19
Glucose, mg/dL	95 (83–119)
Urate, mg/dL	6.2 ± 1.3
Sodium, mEq/L	139 ± 2.6
Potassium, mEq/L	4.3 (3.9–4.6)
Calcium, mg/dL	9.5 ± 0.5
Phosphorus, mg/dL	3.7 ± 0.8
Magnesium, mg/dL	1.7 ± 0.2
Hemoglobin, g/dL	13.2 ± 1.53

**Table 2:** Serum Magnesium (mean ± SD) for the whole cohort and subjects with- and without diabetes at each time point.

	T0	T1	T2
Whole cohort	1.70 ± 0.21	1.81 ± 0.22 **	1.86 ± 0.21 ^‡^
With Diabetes	1.71 ± 0.20	1.84 ± 0.31 *	1.88 ± 0.20 ^ns^
Without Diabetes	1.70 ± 0.21	1.79 ± 0.17 **	1.85 ± 0.21 ^‡^

* *p* < 0.05, ** *p* < 0.01 paired *t*-test T0 vs. T1. ^‡^ *p* < 0.05, ns = not significative; paired *t*-test T1 vs. T2.

**Table 3:** Prevalence of hypomagnesemia at each time point, for the whole cohort ad subject with- and without diabetes.

Time Points	Whole Cohort	With Diabetes	Without Diabetes
T0, *n* (%)	24 (38.1%)	7 (11.1%)	17 (27%)
T1 *, *n* (%)	13 (20.6%) *	5 (7.9%)	8 (12.7%)
T2 **, *n* (%)	10 (15.9%) **	2 (3.2%)	8 (12.7%)

* *p* = 0.019 vs. T0 according to McNemar test for paired data; ** *p* = 0.01 vs. T0 according to McNemar test for paired data.

**Table 4:** Three-month mean (±SD) differences and delta in serum and clinical parameters.

			Delta		
	T1	T2	T1-T0	T2-T0	T2-T1
Body weight, kg	80.6 ± 15.8	78.5 ± 18.3	−0.93 **	−2.6 ^ns^	−1.62 ^ns^
Systolic blood pressure, mmHg	133 ± 13	135 ± 15	−5.87 **	−4.8 *	0.29 ^ns^
Diastolic blood pressure, mmHg	81 ± 9	79 ± 8	−2.87 *	−4.7 **	−2.06 ^ns^
Creatinine, mg/dL	1.55 ± 0.5	1.57 ± 0.6	0.1 *	0.11 ^ns^	0.003 ^ns^
eGFR, mL/min/1.73 m^2^	53 ± 19	53 ± 20	−3.49 *	−3.42 ^ns^	0.27 ^ns^
Urate, mg/dL	5.7 ± 1.3	5.6 ± 1.6	−0.53 *	−0.60 **	−0.09 ^ns^
Sodium, mEq/L	140 ± 2	139 ± 3	0.31 ^ns^	−0.08 ^ns^	−0.33 ^ns^
Calcium, mg/dL	9.6 ± 0.6	9.6 ± 0.6	0.05 ^ns^	0.14 ^ns^	0.01 ^ns^
Phosphorus, mg/dL	3.7 ± 1.1	3.5 ± 0.8	0.07 ^ns^	−0.05 ^ns^	−0.2 ^ns^
Hemoglobin, g/dL	13.4 ± 1.7	13.3 ± 1.8	0.13 ^ns^	0.11 ^ns^	−0.02 ^ns^

*t*-test for paired data; * *p* < 0.05, ** *p* < 0.001, ns = not significative.

## Data Availability

The data are available from the corresponding author (gbilancio@unisa.it) upon reasonable request.

## Abbreviations

The following abbreviations are used in this manuscript:

Mg^2+^	Magnesium
CKD	Chronic kidney disease
CNi	Calcineurin inhibitors
SGLT2i	Sodium-glucose cotransporter 2 inhibitors
KTR	Kidney transplant recipient
eGFR	Estimated glomerular filtration rate
SBP	Systolic blood pressure
DBP	Diastolic blood pressure
IFG	Impaired fasting glucose
